# Latitudinal variation in nematode diversity and ecological roles along the Chinese coast

**DOI:** 10.1002/ece3.2538

**Published:** 2016-10-13

**Authors:** Jihua Wu, Huili Chen, Youzheng Zhang

**Affiliations:** ^1^ Coastal Ecosystems Research Station of the Yangtze River EstuaryMinistry of Education Key Laboratory for Biodiversity Science and Ecological EngineeringSchool of Life SciencesFudan UniversityShanghaiPeople's Republic of China; ^2^ Present address: Hangzhou Key Laboratory for Animal Adaptation and EvolutionHangzhou Normal UniversityHangzhouPeople's Republic of China

**Keywords:** biogeography, dietary niche, feeding selectivity, life‐history group, phylogenetic diversity, soil animal, taxonomic distinctness

## Abstract

**Aim:**

To test changes in the phylogenetic relatedness, niche breadth, and life‐history strategies of nematodes along a latitudinal gradient.

**Location:**

Sixteen wetland locations along the Pacific coast of China, from 20°N to 40°N.

**Methods:**

Linear regression was used to relate nematode phylogenetic relatedness (average taxonomic distinctness (AvTD) and average phylogenetic diversity [AvPD]), life‐history group (based on “*c*‐*p*” colonizer–persister group classification), and dietary specificity (based on guild classification of feeding selectivity) to latitude.

**Results:**

Wetland nematode taxonomic diversity (richness and Shannon diversity indices) decreased with increasing latitude along the Chinese coast. Phylogenetic diversity indices (AvTD and AvPD) significantly increased with increasing latitude. This indicates that at lower latitudes, species within the nematode community were more closely related. With increasing latitude, the nematode relative richness and abundance decreased for selective deposit feeders but increased for nonselective deposit feeders. The proportion of general opportunists decreased with increasing latitude, but persisters showed the opposite trend. The annual temperature range and the pH of sediments were more important than vegetation type in structuring nematode communities.

**Main conclusion:**

Nematode niche breadth was narrower at lower latitudes with respect to dietary specificity. Higher latitudes with a more variable climate favor *r* over *K* life‐history strategists. Nematode communities at lower latitudes contained more closely related species.

## 1 Introduction

Latitudinal gradients in diversity are observed for a wide range of biological groups including plants and animals (Willig, Kaufman, & Stevens, 2003). Dozens of mechanisms have been proposed to explain the pattern of increasing species richness toward low latitudes (Gaston, 2000). Some of the most frequently proposed mechanisms include geographic area, net productivity, species interaction strength, and environmental stability. These mechanisms were used not only to explain changes in species diversity with latitudinal gradient, but also to account for changes in ecological niches. For instance, Krasnov, Shenbrot, Khokhlova, Mouillot, and Poulin (2008) found that the niche breadth of parasitic fleas increased at higher latitudes, both in terms of host specificity and geographic range size. Based on meta‐analysis, González‐Bergonzoni, Meerhoff, Teizeira‐de Mello, Baattrup‐Pedersen, and Jeppesen (2012) reported a global pattern in the relative richness of omnivorous fish species with decreasing latitude. The clutch size of birds was reported to decrease toward the equator in both hemispheres (Cardillo, 2002; Hille & Cooper, 2015). Revealing the latitudinal pattern of ecological niches and roles is important for understanding different ecosystem processes at different latitudes (Vázquez & Stevens, 2004).

Ecological traits of organisms are often linked to phylogenetic history, as closely related species tend to share similar resource requirements and responses to the environment (Wiens et al., 2010). Many low‐latitude clades lack necessary ecological adaptations to survive harsher environments (e.g., low temperature) and cannot cross ecophysiological barriers to unfavorable regions (Ricklefs, 2006). Despite exceptions in polar regions (Adams et al., 2006), coexisting species in a community are often found to be more phylogenetically related at high latitudes where the environment is more dynamic (Qian, Zhang, Zhang, & Wang, 2013). These patterns are mostly proposed for several taxa including plant, bird, and mammal species (Cardillo, Orme, & Owens, 2005; Qian et al., 2013; Safi et al., 2011), and it is still unclear for most taxonomic groups and studies on changes in phylogenetics are far behind those on changes in species richness with latitudinal gradients.

Soil biota consists of a wide range of life forms, which contribute to many essential ecological functions, including decomposition, carbon and nutrient cycling, disease suppression, and regulation of plant growth and primary productivity (Bardgett, Yeates, & Anderson, 2005). Understanding latitudinal patterns in the diversity, ecological niche, and phylogenetic composition of soil biota is important for exploring global patterns of ecosystem functioning (Brussaard et al., 2012). However, the latitudinal patterns of ecological characteristics for belowground biota are more poorly understood and may be different from that of aboveground organisms (Bardgett & van der Putten, 2014; Bardgett et al., 2005; Decaëns, 2010; Wu, Ayres, Bardgett, Wall, & Garey, 2011).

**Figure 1:**
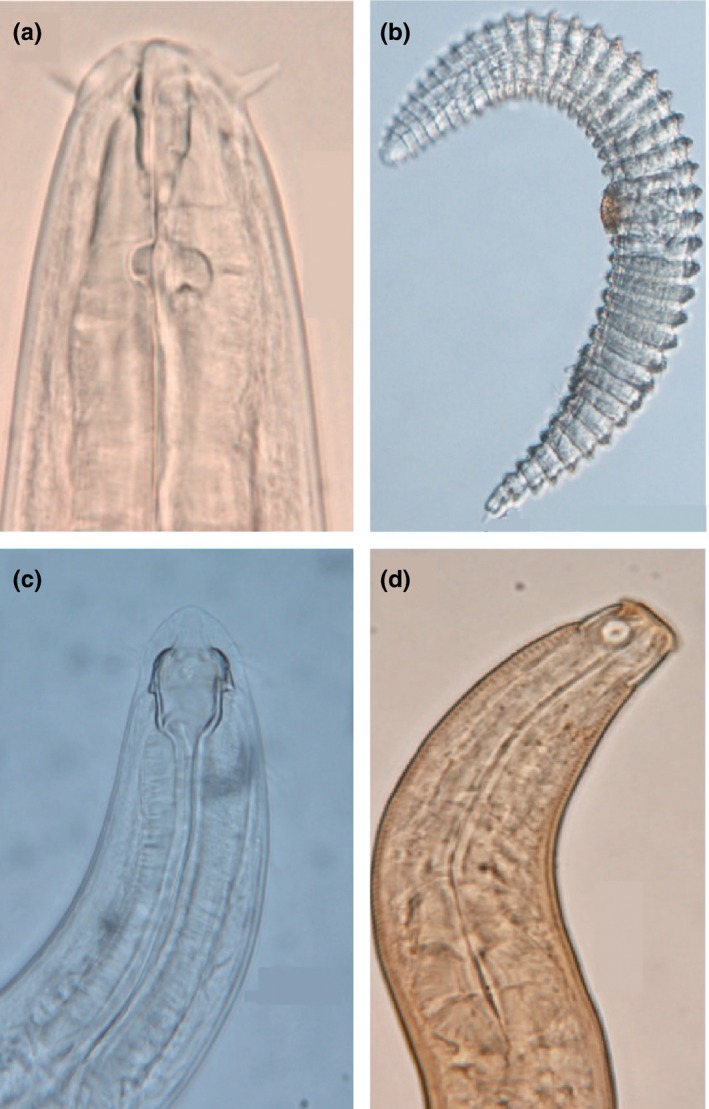
Photographs of some nematode genus in the Chinese coastal wetlands: (a) *Tripyloides*, (b) *Desmoscolex*, (c) *Spaerolaimus*, (d) *Polysigma*

Among soil biota, nematodes hold a central position due to their high abundance and pivotal roles in belowground food webs and ecosystem functioning (Neher, 2010). Reports describing the variations in ecological function and phylogenetic composition of nematode communities across latitudinal gradients are scarce. This study aimed to explore the ecological and evolutionary aspects of nematode diversity distribution across a latitudinal range along the Chinese coast. Using a standardized sampling approach, we collected nematodes (Figure 1) from 16 coastal wetland sites, spanning latitudes from 20°N to 40°N. The relationships of nematode ecological groups (groups with different dietary habits, life‐history strategies, and phylogenetic relatedness) and environmental factors were analyzed. Based on the literature describing these relationships for aboveground taxa, we tested the following hypotheses: (1) nematode taxonomic richness and diversity decrease with increasing latitude, (2) species within communities are more phylogenetically clustered at high latitudes, (3) nematode niche breadth is wider toward high latitudes, and therefore, more dietary generalists are found at high latitudes, and (4) high latitudes are more favorable to large‐bodied *r*‐strategists compared with *K*‐strategists.

## 2 Materials and Methods

### 2.1 Sampling locations and climate data

**Figure 2:**
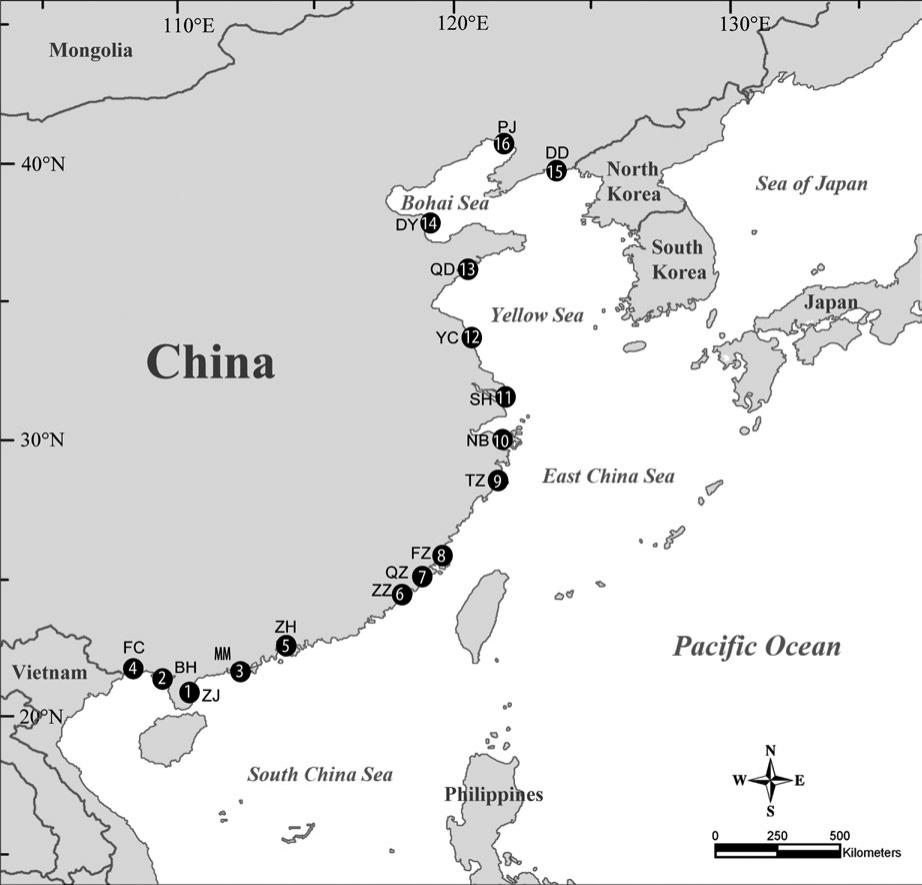
Sampling locations for nematode communities along a latitudinal gradient on the coast of China. Location numbers and abbreviations are listed in Table 1

Sampling was conducted at 16 locations along a latitude ranging from 20.64°N to 40.88°N along the Pacific coast of China (Figure 2). At each location, several (1–7) marsh or mangrove wetlands spaced more than 500 m apart were selected (Table 1), forming a total of 53 wetlands. Intertidal marsh habitats were dominated by *Phragmites australis* or *Suaeda salsa*, the most common salt marsh plants on the coast of China. At the more southern sampling locations, the dominant mangroves included *Kandelia candel*,* Avicennia marina*,* Aegiceras corniculatum,* and *Acanthus ilicifolius*.

**Table 1:** Sampling locations showing latitude, vegetation type, and the number of samples collected

Location number	Location	Location abbreviation	Latitude	Vegetation type	Samples
1	Zhanjiang	ZJ	20.64°N	Mangrove	2
2	Beihai	BH	21.50°N	Mangrove	3
3	Maoming	MM	21.51°N	Mangrove	3
4	Fangcheng	FC	21.62°N	Mangrove	3
5	Zhuhai	ZH	22.43°N	Mangrove	2
				Marsh_‐*Phragmites*_	2
6	Zhangzhou	ZZ	24.44°N	Mangrove	4
7	Quanzhou	QZ	24.95°N	Mangrove	1
8	Fuzhou	FZ	26.06°N	Marsh_‐*Phragmites*_	3
9	Taizhou	TZ	28.86°N	Marsh_‐*Phragmites*_	3
10	Ningbo	NB	29.96°N	Marsh_‐*Phragmites*_	3
11	Shanghai	SH	31.55°N	Marsh_‐*Phragmites*_	2
12	Yancheng	YC	33.81°N	Marsh_‐*Phragmites*_	3
13	Qingdao	QD	36.19°N	Marsh_‐*Suaeda*_	3
14	Dongying	DY	37.72°N	Marsh_‐*Suaeda*_	3
				Marsh_‐*Phragmites*_	4
15	Dandong	DD	39.88°N	Marsh_‐*Phragmites*_	3
16	Panjin	PJ	40.88°N	Marsh_‐S*uaeda*_	3
				Marsh_‐*Phragmites*_	3

Climate data for each locality including annual temperature (AT), annual temperature range (ATR), and annual precipitation (AP) were obtained from the WORLDCLIM dataset, version 1.3 (www.worldclim.org), using the Bloclim extension on DIVA‐GIS 5.2. The normalized difference vegetation index (NDVI) was obtained from http://glcf.umiacs.umd.edu/data/gimms/.

### 2.2 Sampling and sediment characteristics

Sampling was carried out between August and September 2007, when productivity was highest in the year. Sediment samples were collected using a modified O'Connor split corer (3.2 cm diameter) to 10 cm depth. At each of the 53 wetlands from 16 sampling localities, five sediment cores were collected at 2‐m intervals along an 8‐m transect. Samples were combined together to form a composite sample. Sediment samples were homogenized and then split into two parts. About 150 g of sediment was fixed in 4% hot formalin for nematode analysis. The remainder of the sample was air‐dried and analyzed for sediment grain size composition, total carbon, total nitrogen, and pH. Sediment grain size was measured using a vibrating sieve shaker (FRITSCH analysette 3, Germany). Total carbon and total nitrogen was measured using a NC Soil Analyzer (Flash EA 11121 Series, Thermo Finnigan, Italy). The pH value was measured using a pH meter (IQ150, USA).

### 2.3 Nematodes

Nematodes were extracted by flotation in Ludox TM in the laboratory. After counting the total number of nematode individuals, about 120 specimens per sample were randomly selected for identification. Nematodes were identified to morphospecies (morphologically distinct taxa) level following Goodey (1963), Jairajpuri and Ahmad (1992), and Warwick, Platt, and Smoerfield (1998). For simplicity, the morphospecies were referred as “species” hereafter. The Shannon–Weaver diversity index *H′* = Σ*p*_*i*_ ln *p*_*i*_, where *p*_*i*_ is the proportion of individuals in the *i*th taxon, was calculated at the species and genus level.

Based on characteristics of the buccal cavity, nematodes were identified as plant feeders or free‐living nematodes. Free‐living nematodes were further classified into four groups based on feeding types (Wieser, 1953): (1) selective deposit feeders which have no buccal cavity or only a narrow tubular buccal cavity, (2) nonselective deposit feeders which have a large buccal cavity without teeth, (3) epistrate or diatom feeders which have a buccal cavity armed with teeth, and (4) predators or omnivores which have large teeth or jaws.

Based on life history of nematodes at the family level, nematodes were designated a colonizer–persister (*c*‐*p*) value ranging from 1 to 5 (Bongers, 1990). Nematodes with low *c‐p* values have a short generation time and high fecundity and are comparable to *r‐*strategies in the loose sense (Bongers, 1990). On the other hand, nematodes with high *c‐p* values have a long generation time, large body size and low fecundity and are sensitive to disturbance and can be considered as *K‐*strategies (sensu lato) (Bongers, 1990). Nematodes were classified into one of three categories: enrichment opportunists (*c‐p* 1), general opportunists (*c‐p* 2), and persisters (*c‐p* 3‐5) (De Goede, Bongers, & Ettema, 1993).

The proportion of species representing “relative species richness” and the proportion of individuals representing “relative abundance” for each nematode ecological group were calculated. The proportion of species was calculated as *S*_*i*_/*S*_*t*_, where *S*_*i*_ is the species richness of a certain ecological group within a community, and *S*_*t*_ is the total species richness of the whole community. The proportion of individuals of each group was calculated as *I*_*i*_/*I*_*t*_, where *I*_*i*_ is the individual number of a certain ecological group within a community, and *I*_*t*_ is the total number of individuals in the whole community.

To assess the phylogenetic composition of a certain nematode community, two indices, average taxonomic distinctness (AvTD) and average phylogenetic diversity (AvPD), were selected. AvTD is a distance‐based index calculated using pairwise distance. A higher value of AvTD or AvPD indicates a larger phylogenetic relationship among taxa. According to Schweiger, Klotz, Durka, and Kuehn (2008), AvTD is suggested as the best index to compare independent communities because it is unbiased by species richness. AvPD represents a distance‐based index and uses a minimum spanning path to reflect phylogenetic skewness.

Average taxonomic distinctness (AvTD) (Clarke & Warwick, 1998) is a measure of the average degree to which species in an assemblage are related to each other. AvTD = [Σ Σ_*i*<*j*_ ω_*ij*_]/[*s*(*s*–1)/2], where *s* is the number of species present, the double summation is over {*i *=* *1, … *s*;* j *=* *1, … *s*, such that *i *< *j*}, and ω_*ij*_ is the “distinctness weight” given to the path length linking species *i* and *j* in the hierarchical classification. The taxonomic levels used in this study are species, genus, family, order, and class, according to the classification described by Lorenzen (1981). Values of AvTD are based on equal step lengths between the above five taxonomic levels. Thus, the weighting between taxonomic levels for different species of the same genus is ω = 20, for species in the same family but different genera ω = 40, for species in the same order but different families ω = 60, and for species in different classes ω = 100.

Average phylogenetic diversity (AvPD) (Clarke & Warwick, 2001) is a measure of the average amount of phylogenetic diversity (branch length) contributed by randomly chosen species to the total phylogenetic diversity (PD). AvPD = PD/*s *= Σ*n*_*i*_/*s*, where *s* is the number of species present, and *n*_*i*_ is the number of i nodes within the minimum spanning path.

### 2.4 Data analyses

Linear regression was used to determine to what degree nematode richness, *H′* diversity indices, feeding type, and colonizer–persister (*c‐p*) groups could be explained by latitude. To examine the relationship between nematode variables and indices and environmental factors, model selections in regression analyses using Akaike's information criterion (AIC) corrected for small sample size (AIC_C_) were performed. Environmental factors considered in models included annual temperature (AT), annual temperature range (ATR), annual precipitation (AP), the yearly normalized difference vegetation index (NDVI), total nitrogen (TN), total carbon (TC), percentage sand, and pH. AIC_c_ weight (wAIC_c_) and *R* ^2^ values for each model were calculated. Akaike weight (wAIC_c_) represents the probability that the model is the best model among the given set of alternatives. *R* ^2^ value represents the measure of the model's goodness of fit. Pearson coefficients were calculated for investigating correlations between nematodes and environmental parameters (climate factors and physiochemical characteristics of sediment). The analyses were executed using the statistical package Statistica (version 6.0, StatSoft Inc, Tulsa OK, USA). Both AIC model selection and Pearson coefficients were calculated only for nematode variables and indices displaying a significant latitudinal gradient.

To examine geographic patterns in nematode community structure, ordination plots were produced by nonmetric multidimensional scaling (MDS) using a ranked similarity matrix based on Bray–Curtis similarity measures of nematode communities from 16 locations representing three vegetation types. Multivariate differences in nematode community composition for all samples were analyzed using Biota‐Environment matching (BIO‐ENV). Both analyses were performed using Primer v5.0 (Primer‐E Ltd., Plymouth, UK). The proportional data were log(*x* + 1)‐transformed prior to MDS and BIO‐ENV analyses. The analyses were undertaken using the PRIMER (Plymouth Routines In Multivariate Ecological Research) version 5.2 software package.

## 3 Results

**Figure 3:**
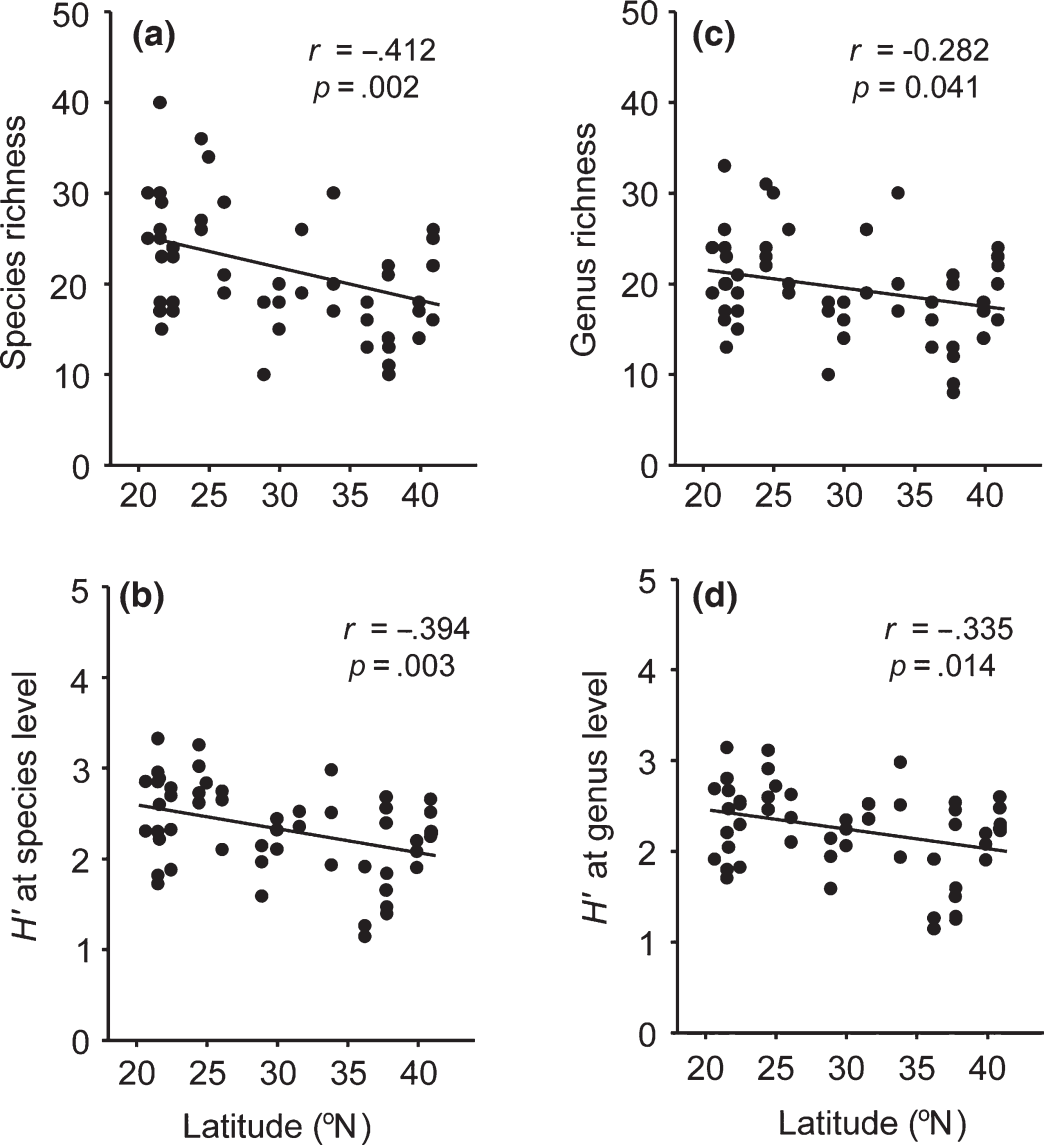
Relationship between taxonomic diversity indices and latitude for (a) species richness, (b) *H′* at species level, (c) genus richness, and (d) *H′* at genus level. *n *= 53

A total of 166 nematode morphospecies were collected from 16 locations belonging to 112 genera, 44 families, 7 orders, and 2 classes (Appendix S1). Richness and *H′* diversity indices of nematodes at both the species level and genus level significantly decreased with increasing latitude (Figure 3). This indicates that southern localities had a greater diversity of nematodes compared with northern locations.

**Figure 4:**
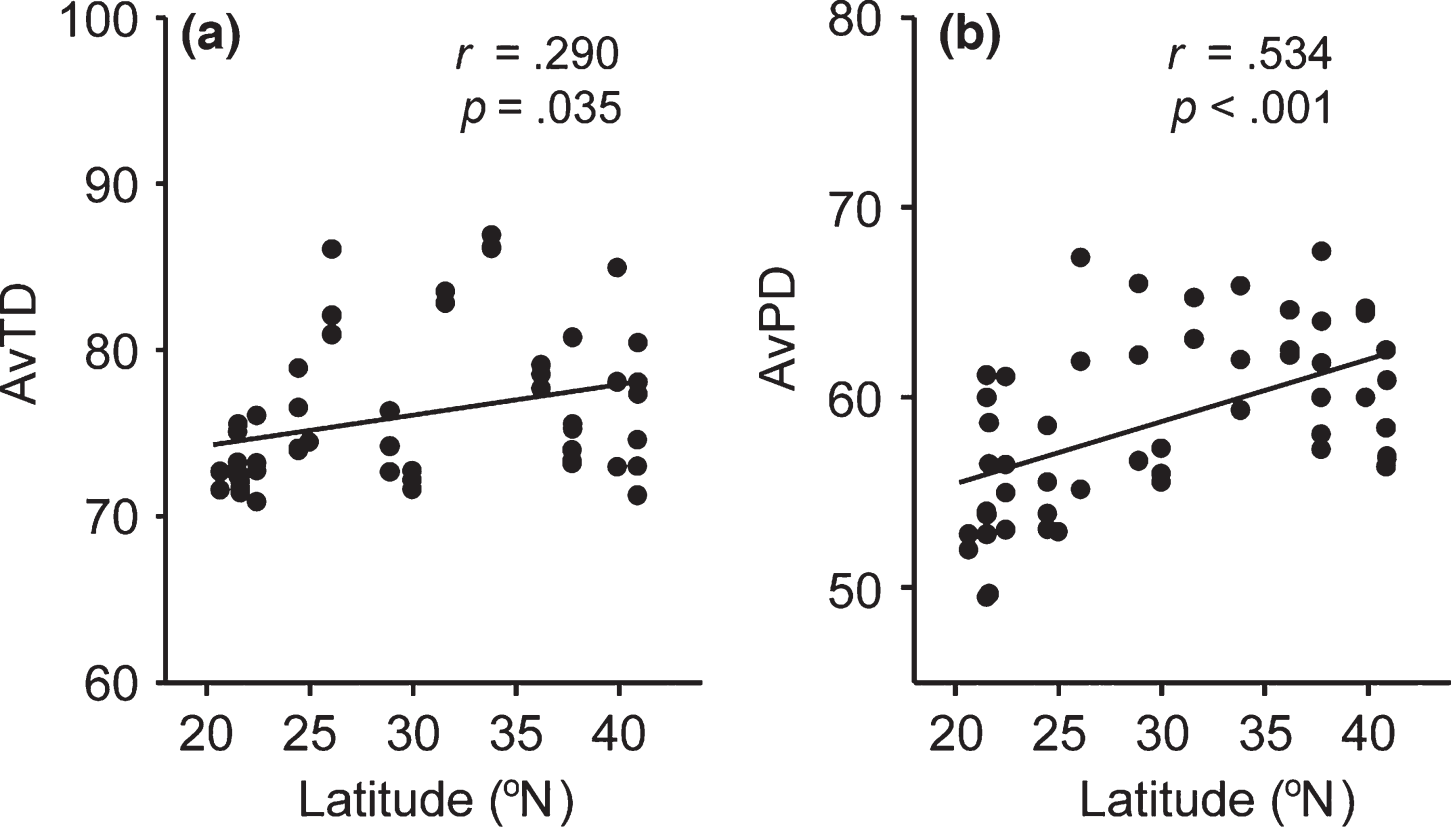
Relationship between phylogenetic diversity indices and latitude for (a) average taxonomic distinctness (AvTD) and (b) average phylogenetic diversity (AvPD). *n *=* *53

Phylogenetic diversity indices (AvTD and AvPD) significantly increased with increasing latitude (Figure 4). This indicates that the nematode communities at the lower latitudinal locations contained more closely related species.

**Figure 5:**
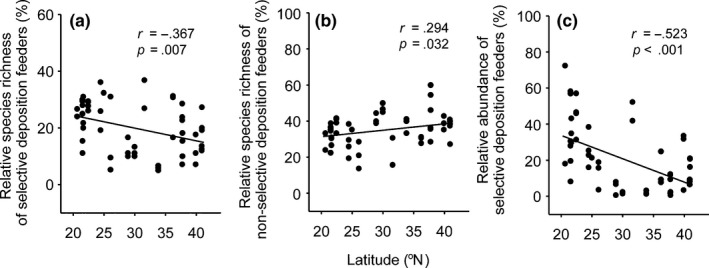
Relationship between latitude and different nematode feeding groups for (a) relative species richness of selective deposit feeders, (b) relative species richness of nonselective deposit feeders, and (c) relative abundance of selective deposit feeders. *n *=* *53

The proportion of selective deposit feeders was negatively related to increasing latitude, for both species and individuals (Figure 5a,c). However, the proportion of nonselective deposit feeders showed a weak positive relationship with increasing latitude for species (Figure 5b). The proportion of individual nonselective deposit feeders also showed a positive relationship with increasing latitude that was statistically marginally significant (*r* = .266, *p *=* *.054, data not shown). Significant latitudinal gradients were not detected for other trophic groups including plant feeders, epistrate or diatom feeders, and predators or omnivores (data not shown).

**Figure 6:**
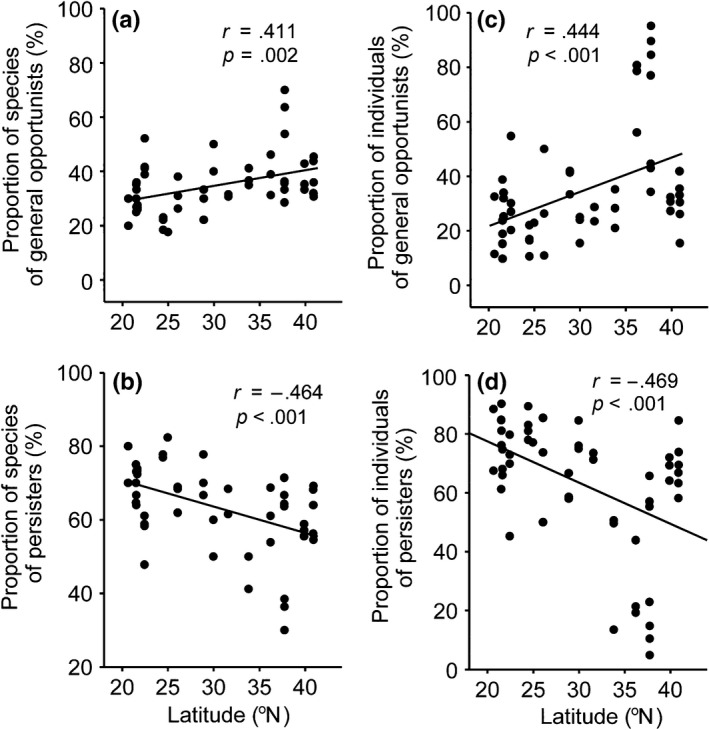
Relationship between the proportion of species or individuals from each colonizer–persister (*c*‐*p*) group and latitude (^o^N) for (a) proportion of general opportunist (*c*‐*p* 2) species, (b) proportion of persister (*c*‐*p* 3‐5) species, (c) proportion of general opportunist (*c*‐*p* 2) individuals, (d) proportion of persister (*c*‐*p* 3‐5) individuals. *n *=* *53

The proportion of nematode species and individuals that were classified as general opportunists (*c‐p* 2) showed a significant increase with increasing latitude. However, the proportion of species and individuals that were classified as persisters (*c‐p* 3‐5) showed the opposite trend (Figure 6). No significant relationships between nematodes classified as enrichment opportunists (*c‐p* 1) and latitude were observed (data not shown).

The climatic parameters annual temperature (AT) and annual temperature range (ATR) were the most frequently selected parameters in models developed to investigate variables that may be associated with nematode distribution (Appendix S2). The normalized difference vegetation index (NDVI), which represents vegetation and soil condition measures, was relatively a weak predictor. Correlation coefficient analyses showed that the taxonomic diversity of nematodes (richness and *H′* at both the species level and generic level) were positively correlated with AT, but negatively correlated with ATR and pH (Table 2). The phylogenetic diversity indices (AvTD and AvPD) were both negatively correlated with AT, but positively correlated with ATR and pH. The proportion of nematodes that were classified as selective deposit feeders and as persisters (*c*‐*p* 3‐5) was positively associated with AT and precipitation and negatively associated with ATR and pH (Table 2). Conversely, the proportion of nonselective deposit feeders and general opportunists (*c*‐*p* 2) exhibited the opposite pattern (Table 2).

**Table 2:** Correlation coefficients of taxonomic richness and diversity, phylogenetic diversity, relative abundance, and richness of different feeding groups and colonizer–persister groups with climatic parameters and physiochemical parameters of soil (AT: annual temperature; ATR: annual temperature range; AP: annual precipitation; NDVI: yearly normalize difference vegetation index; TN %: total nitrogen; TC %: total carbon; sand %; pH). Significance levels are marked by asterisk (*p < .05, **p < .01,***p < .001)

	AT	ATR	AP	NDVI	Sand %	TC %	TN %	pH
	r	r	r	r	r	r	r	r
*Taxonomic diversity*								
Genus number	0.31*	−0.26	0.16	0.03	−0.05	−0.05	0.14	−0.21
Species richness	0.43**	−0.39**	0.21	0.02	0.01	−0.05	0.15	−0.33*
*H*’ at genus level	0.35*	−0.30*	0.27	−0.01	0.06	0.03	0.18	−0.25
*H*’ at species level	0.41**	−0.37**	0.28	−0.02	0.13	0.06	0.17	−0.32*
*Phylogenetic diversity*								
AvTD	−0.29*	0.29*	−0.14	−0.03	−0.07	0.03	−0.05	0.30*
AvPD	−0.54***	0.52***	−0.28	−0.02	−0.04	0.07	−0.16	0.43**
*Relative species richness of ecological groups*								
selective deposit feeders	0.42**	−0.39**	0.32*	0.07	0.18	0.06	0.24	−0.33*
nonselective deposit feeders	−0.33*	0.31*	−0.31*	−0.18	−0.08	0.02	−0.17	0.41**
*c‐p* 2 (general opportunists)	−0.42**	0.40**	−0.36*	0.01	−0.02	0.27	0.03	0.41**
*c‐p* 3‐5 (persisters)	0.47**	−0.45**	0.37**	0.05	0.04	−0.26	0.03	−0.46**
*Relative abundance of ecological groups*								
selective deposit feeders	0.56***	−0.53***	0.57***	0.05	0.12	0.06	0.39**	−0.60***
*c‐p* 2 (general opportunists)	−0.44**	0.39**	−0.43**	0.03	0.06	0.11	−0.24	0.40**
*c‐p* 3‐5 (persisters)	0.47**	−0.42**	0.45**	0.06	−0.03	−0.13	0.30*	−0.47**

Based on the nonmetric MDS plots, the 16 sampling sites were separated into two groups (Appendix S3). One group included the nematode communities from the southern locations (sites 1 to 8, 20.64°N to 26.06°N), distributed along the coast of South China Sea. The other group included nematodes from the northern locations (sites 9 to 16, 28.87°N to 40.88°N), distributed along the coast of the East China Sea, the Yellow Sea, and the Bohai Sea. Nematode communities representing the three vegetation types (mangroves, *Phragmites australis* marsh, and *Suaeda salsa* marsh) overlapped each other and could not be discriminated clearly (Appendix S3). BIO‐ENV analyses indicated that annual temperature range (ATR) was strongly correlated with nematode community structure, followed by pH and NDVI (Appendix S4). The best combination of environmental variables explaining nematode community structure included ATR and pH, with a correlation coefficient of 0.615.

## 4 Discussion

### 4.1 Nematode richness, diversity, and community structure

Studies based on synthesized data of nematode diversity mainly yielded two different findings. Some studies concluded that nematode diversity increased with increasing latitude (e.g., Boucher, 1990; Boucher & Lambshead, 1995; Procter, 1984), and others concluded that there were no clear trends in the diversity of nematodes with changing latitude (e.g., Boag & Yeates, 1998; Fonseca & Netto, 2015; Mokievsky & Azovsky, 2002). These contradictory findings may result from unbalanced sampling effort and taxonomic resolution on nematodes from different latitudes (Lee & Riveros, 2012). Therefore, a full understanding of latitudinal patterns of nematode distribution on a global scale may require a more complete taxonomic knowledge and molecular information of this highly diversified group (Wu et al., 2011).

Most studies based on field samplings found that nematode diversity decreased with increasing latitude (Lambshead et al., 2002; Lee & Riveros, 2012; Nicholas & Trueman, 2005; Nielsen et al., 2014), which is consistent with our results. However, different conclusions were drawn by Lambshead, Tietjen, Ferrero, and Jensen (2000) and Gobin and Warwick (2006). Lambshead et al. (2000) found that species richness of deep‐sea nematodes increased along a latitude gradient. However, their findings may be confounded by sampling depth (Rex, Stuart, & Etter, 2001). Gobin and Warwick (2006) did not find a clear trend in nematode diversity with change in latitude. Given that they collected nematodes using artificial collectors at only four geographic locations, their results may differ from studies that directly sample the soil or sediment. Therefore, in terms of diversity per unit area, the pattern of higher species richness at lower latitudes may hold true for nematodes, similar to the majority of other organisms. The diversity of ants was positively correlated with annual temperature and negatively correlated with temperature range (Dunn et al., 2009). Similarly, we found that nematode richness and diversity in coastal wetlands were positively correlated with annual temperature and negatively correlated with temperature range.

Fonseca and Netto (2015) discovered that nematode species composition differed significantly between estuaries with and without mangroves. They speculated that differences in root systems and leaf decomposition processes between mangrove estuaries and salt marshes shaped the different nematode communities. Other studies also have reported the importance of vegetation type on nematode community composition (Nielsen et al., 2014). However, in our study, the effect of vegetation type on the nematode community was not clear, probably because plant‐feeding nematodes in coastal wetlands are not as abundant and diverse in terrestrial habitats. MDS analyses showed that at latitudes of less than 26°N, nematode communities in mangrove habitats and *P. australis* marshes could not be distinguished. At more northern locations (latitudes of 28°N or higher), nematode communities from marshes dominated by *P. australis* and *S. salsa* also overlapped. Based on BIO‐ENV analyses, our study showed that climate variables, such as annual temperature range (ATR) and sediment properties such as pH, were more important than the dominant vegetation type.

### 4.2 Nematode phylogenetic diversity

Negative relationships between increasing latitude and phylogenetic diversity have been reported for angiosperm plants (Qian et al., 2013), mammals (Safi et al., 2011), etc. Phylogenetically closely related species tend to share similar habitat requirements and thus are less likely to coexist if the community is structured by competition (Graham, Parra, Rahbek, & Mcguire, 2009; Ulrich & Fattorini, 2013). However, our results showed a positive relationship between nematode phylogenetic diversity (both average taxonomic distinctness AvTD and average phylogenetic diversity AvPD) and increasing latitude. This pattern is probably because small organisms or soil biota are weakly structured by competition (Decaëns, 2010; Ulrich & Fattorini, 2013).

Species living in harsh environments are reported to be more phylogenetically clustered (Graham et al., 2009). This may be because few clades can across ecophysiological barriers from benign environments to harsher ones (e.g., colder or with more disturbance) (Helmus et al., 2010). However, our results indicate that nematodes are phylogenetically more dispersed in colder regions with more variable climates (Table 2). These inconsistencies may result from other biotic interactions such as facilitation or the mobility of organisms that can also affect phylogenetic structure along a stressful habitat gradient (Graham et al., 2009). The exceptions in Antarctica (taxa formed from a wide phylogenetic base) also suggest that it may be related to the possible endemicity and broad dispersal of small‐sized soil organisms (Adams et al., 2006, 2007). Pattern of nematode community assembly, speciation, extinction with latitudes needs further investigation by utilizing phylogenetic information.

### 4.3 Nematode dietary breadth

A positive relationship between niche breadth and latitude has long been assumed (Morin & Chuine, 2006). According to the pervasive “climate variability hypothesis,” greater fluctuations in climate at high latitudes cause more variability in resource availability in time and space and thus favor wider niches (Cardillo, 2002; Slove & Janz, 2010). By studying the feeding plasticity of birds, Simon, Diamond, and Schwab (2003) found that Canadian southern forests were dominated by specialists and northern forests by generalists. Krasnov et al. (2008) found that in terms of host specificity, the niche breadth of parasitic fleas on mammals increased with latitude. Dietary specialization determines an organism's resource base and constitutes an important part of niche breadth. In this study, we analyzed selective and nonselective groups of deposit feeding nematodes separately. Selective‐feeding nematodes are considered to be more specialized in food (certain groups of bacteria or organic matter) uptake and therefore have a narrower dietary niche breadth. Our results showed that nonselective‐feeding nematodes were more abundant at higher latitudes, whereas selective feeders showed the opposite pattern. This may also be because food availability is more variable at higher latitudes where there are greater fluctuations in climate. Feeding selectivity of nematodes could lead to a shift in microbial composition and exert cascading effects on ecosystem processes. Nematode groups with different dietary specializations exhibited different latitudinal patterns, which may suggest possible differences in microbial composition, nutrient cycling rates, and ecosystem vulnerability in different geographic regions.

### 4.4 Nematodes with different life‐history strategy

Latitude‐related variability in the environment not only influences resource use (e.g., dietary specialization), but may also alter the responses of organisms to the environment. Although the ecological traits of *K*‐ and *r*‐strategists were not directly measured in this study, the classification of nematodes along the colonizers (*c*) to persisters (*p*) spectrum reflects some typical characteristics of life‐history responses. We found that the relative abundance and richness of general opportunists (*c*‐*p* 2) increased with increasing latitude, lower annual temperature, and a higher temperature range. Nematodes that were classified as persisters (*c*‐*p* 3‐5) and showed typical *K*‐selected characteristics decreased in relative abundance and richness with increasing latitude. Studies of other taxonomic groups support our findings that *K*‐strategists may be more common at lower latitudes. For instance, birds from low latitudes have been found to possess *K*‐strategy characteristics such as smaller clutch size, large eggs, reduced mortality, and higher survival (Cardillo, 2002; Hille & Cooper, 2015). For plants, a trend of increasing seed size toward the tropics has been reported (Morin & Chuine, 2006). However, the generalization that more *K*‐strategists occur at lower latitudes is still under debate (Auer & King, 2014). High‐latitude gammaridean amphipod species were characterized by large body size, long generation, few broods, large embryos which showed *K*‐selected life‐history strategy (Sainte‐Marie, 1991). In Europe, higher‐latitude populations of freshwater fish have been found grew more slowly, matured later, had a longer life span than lower‐latitude populations (Blanck & Lamouroux, 2007). High‐latitude coastal reefs are found to be typified by stress‐tolerant generalist coral species (Sommer, Harrison, Beger, & Pandolfi, 2014). For nematode, the highly abundant nematode species *Scottnema lindsayae* in Antarctic continent soils has a long lifecycle (218 days) and development time, low fecundity, which more closely resembles a *K*‐selective life‐history strategy (Adams et al., 2007). The pattern observed in our study may be explained as high local climate variability at higher latitudes benefits *r*‐strategists, which may recover more easily from disturbance (Gaston, 2000; Hille & Cooper, 2015). However, the true underlying mechanism needs further investigation.

Both large‐scale climatic variables and small‐scale soil parameters could be determinants in structuring nematode community. In addition to the variables including annual temperature, annual temperature range, and soil pH which were significantly associated with nematode diversity, annual precipitation and total soil nitrogen were also found relating to the relative abundance and species richness of nematode ecological groups. This suggests that a wider range of factors may be associated with variations among nematode ecological groups compared with the number of factors that influence nematode species diversity.

## 5 Conclusion

Information on the geographic distribution of nematodes is scarce for the Asian region. This is the first study to examine the association between nematode diversity and latitude with respect to dietary specialization and life‐history strategies. In relation to our initially proposed hypotheses, we conclude the following: (1) The relative richness and abundance of nematodes that were dietary specialists tend to be greater at lower latitudes. (2) Nematodes with *r* life histories were favored over *K* life‐history strategists at higher latitudes, where the climate was more variable. (3) Nematode species within a community tended to be more closely related in phylogeny at lower latitudes, which may imply that these communities were weakly structured by competition. (4) Wetland nematode diversity decreased with increasing latitude from 20°N to 40°N along the Chinese coast, and annual temperature range and soil pH were more important than vegetation type in influencing nematode community structure.

## Conflict of interest

None declared.
