# Supplementary material for: Latitudinal variation in nematode diversity and ecological roles along the Chinese coast
Source: Ecol Evol. 2016 Oct 13;6(22):8018–27. doi: 10.1002/ece3.2538 (PMC5108254; doi:10.1002/ece3.2538)
Supplement: Supplementary file 1 [file ECE3-6-8018-s001.docx]

**SUPPORTING INFORMATION**

**Article title: Latitudinal variation in nematode diversity and ecological roles along the Chinese coast**

Authors: Jihua Wu^*^, Huili Chen, Youzheng Zhang

Appendix S1 List of nematode taxa encountered at each location (shown are genera level), feeding type and colonizer-persister values. The number of each sampling location is described in Table 1 and Figure 2. Abbreviation of feeding type Pl: plant feeders; 1A: selective deposit feeders which have no buccal cavity or only a narrow tubular buccal cavity; 1B: non-selective deposit feeders which have a large buccal cavity without teeth; 2A: epistrate or diatom feeders which have a buccal cavity armed with teeth; 2B: predators or omnivores which have large teeth or jaws.

| Nematode taxa | | | | Location Number | | | | | | | | | | | | | | | | Feeding  type | *c-p*  value |
| --- | --- | --- | --- | --- | --- | --- | --- | --- | --- | --- | --- | --- | --- | --- | --- | --- | --- | --- | --- | --- | --- |
|  |  |  |  | 1 | 2 | 3 | 4 | 5 | 6 | 7 | 8 | 9 | 10 | 11 | 12 | 13 | 14 | 15 | 16 |  |  |
| Class ADENOPHOREA | | | |  |  |  |  |  |  |  |  |  |  |  |  |  |  |  |  |  |  |
|  | Order CHROMADORIDA | | |  |  |  |  |  |  |  |  |  |  |  |  |  |  |  |  |  |  |
|  |  | Family AEGIALOALAIMIDAE | |  |  |  |  |  |  |  |  |  |  |  |  |  |  |  |  |  |  |
|  |  |  | *Aegialoalaimus* |  | √ |  |  |  | √ | √ |  |  |  |  |  |  |  |  |  | 1A | 2 |
|  |  |  | *Southernia* |  |  |  |  |  |  |  |  |  |  |  |  |  |  |  | √ | 1A | 2 |
|  |  | Family CHROMADORIDAE | |  |  |  |  |  |  |  |  |  |  |  |  |  |  |  |  |  |  |
|  |  |  | *Chromadorella* |  | √ | √ | √ |  |  |  |  |  |  |  |  |  |  |  |  | 2A | 3 |
|  |  |  | *Chromadorina* |  |  | √ |  |  |  |  |  |  |  |  |  |  |  |  |  | 2A | 3 |
|  |  |  | *Chromadorita* |  | √ | √ |  | √ |  |  |  |  | √ | √ |  |  |  | √ |  | 2A | 3 |
|  |  |  | *Hypodontolaimus* |  | √ | √ | √ | √ | √ | √ | √ | √ | √ | √ | √ |  | √ | √ | √ | 2A | 3 |
|  |  |  | *Innocuonema* |  |  |  |  |  |  |  |  |  |  |  | √ | √ |  |  |  | 2A | 3 |
|  |  |  | *Neochromadora* |  |  |  |  |  |  |  |  |  |  |  |  | √ |  | √ | √ | 2A | 3 |
|  |  |  | *Ptycholaimellus* | √ | √ | √ | √ | √ | √ |  | √ | √ | √ | √ | √ | √ | √ | √ | √ | 2A | 3 |
|  |  | Family CHRONOGASTERIDAE | |  |  |  |  |  |  |  |  |  |  |  |  |  |  |  |  |  |  |
|  |  |  | *Chronogaster* |  |  |  |  |  |  |  | √ |  |  |  |  |  |  |  |  | 1B | 2 |
|  |  | Family COMESOMATIDAE | |  |  |  |  |  |  |  |  |  |  |  |  |  |  |  |  |  |  |
|  |  |  | *Dorylaimopsis* | √ | √ | √ |  |  | √ |  |  |  |  |  |  |  |  |  |  | 2A | 3 |
|  |  |  | *Hopperia* |  | √ |  |  |  | √ | √ |  |  |  |  |  |  |  |  |  | 1B | 3 |
|  |  |  | *Pierrickia* |  |  |  |  |  |  |  |  |  |  | √ |  |  |  |  |  | 1A | 3 |
|  |  |  | *Sabatieria* | √ | √ | √ | √ | √ | √ | √ |  | √ | √ |  |  |  |  |  |  | 1B | 3 |
|  |  | Family CYATHOLAIMIDAE | |  |  |  |  |  |  |  |  |  |  |  |  |  |  |  |  |  |  |
|  |  |  | *Longicyatholaimus* |  |  |  | √ |  |  |  |  |  |  |  |  |  |  |  |  | 2A | 3 |
|  |  |  | *Marylynnia* | √ | √ | √ | √ |  | √ |  |  |  |  |  |  |  |  |  |  | 2A | 3 |
|  |  |  | *Paracanthonchus* |  |  |  |  | √ | √ |  | √ | √ | √ | √ | √ | √ | √ | √ | √ | 2A | 3 |
|  |  |  | *Paracyatholaimus* | √ |  | √ |  |  |  |  |  |  |  |  |  |  |  |  |  | 2A | 3 |
|  |  | Family DESMODORIDAE | |  |  |  |  |  |  |  |  |  |  |  |  |  |  |  |  |  |  |
|  |  |  | *Chromaspirina* |  | √ |  |  |  | √ |  |  |  |  |  |  |  |  |  |  | 2A | 3 |
|  |  |  | *Desmodora* | √ | √ |  | √ | √ | √ | √ | √ | √ | √ |  |  |  | √ |  | √ | 2A | 3 |
|  |  |  | *Metachromadora* | √ | √ |  | √ |  | √ |  |  | √ | √ |  |  |  | √ |  |  | 2A | 3 |
|  |  | Family DESMOSCOLECIDAE | |  |  |  |  |  |  |  |  |  |  |  |  |  |  |  |  |  |  |
|  |  |  | *Calligyrus* |  |  |  |  |  |  |  |  |  |  |  |  |  |  |  | √ | 1A | 4 |
|  |  |  | *Desmoscolex* |  | √ | √ |  |  | √ | √ | √ |  |  | √ |  | √ | √ |  | √ | 1A | 4 |
|  |  |  | *Pareudesmoscolex* |  |  |  |  |  |  |  | √ |  |  |  |  |  |  |  |  | 1A | 4 |
|  |  |  | *Quadricoma* | √ | √ |  | √ | √ | √ |  | √ |  |  | √ |  |  |  |  |  | 1A | 4 |
|  |  |  | *Spirinia* | √ | √ |  | √ |  | √ |  |  |  |  |  |  |  |  |  |  | 1A | 4 |
|  |  | Family ETHMOLAIMIDAE | |  |  |  |  |  |  |  |  |  |  |  |  |  |  |  |  |  |  |
|  |  |  | *Comesa* |  | √ |  | √ |  |  |  |  |  |  |  |  |  |  |  |  | 2A | 3 |
|  |  |  | *Ethmolaimus* |  |  |  | √ | √ | √ | √ | √ |  |  |  |  |  | √ |  |  | 2A | 3 |
|  |  | Family HALIPLECTIDAE | |  |  |  |  |  |  |  |  |  |  |  |  |  |  |  |  |  |  |
|  |  |  | *Haliplectus* | √ |  | √ | √ |  | √ |  | √ |  |  | √ |  | √ |  |  | √ | 1A | 3 |
|  |  | Family LEPTOLAIMIDAE | |  |  |  |  |  |  |  |  |  |  |  |  |  |  |  |  |  |  |
|  |  |  | *Antomicron* |  |  |  |  |  | √ | √ |  |  |  |  |  |  | √ |  |  | 1A | 3 |
|  |  |  | *Camacolaimus* | √ | √ | √ | √ |  | √ |  |  |  |  |  |  |  |  | √ | √ | 1B | 3 |
|  |  |  | *Deontolaimus* |  |  |  |  |  |  |  |  |  |  |  | √ |  |  |  | √ | 1B | 3 |
|  |  |  | *Leptolaimoides* |  | √ |  |  |  |  |  | √ |  |  |  |  | √ |  |  |  | 1A | 2 |
|  |  |  | *Leptolaimus* |  | √ | √ |  | √ | √ | √ | √ |  | √ | √ |  |  | √ | √ | √ | 1A | 2 |
|  |  |  | *Procamacolaimus* |  |  |  | √ |  |  |  |  |  |  |  |  |  |  |  |  | 1B | 2 |
|  |  | Family MICROLAIMIDAE | |  |  |  |  |  |  |  |  |  |  |  |  |  |  |  |  |  |  |
|  |  |  | *Microlaimus* | √ | √ | √ | √ |  | √ |  |  |  |  |  |  |  | √ |  | √ | 2A | 2 |
|  |  | Family SELACHINEMATIDAE | |  |  |  |  |  |  |  |  |  |  |  |  |  |  |  |  |  |  |
|  |  |  | *Halichoanolaimus* | √ | √ | √ | √ |  | √ |  |  |  |  |  |  |  |  |  | √ | 2B | 3 |
|  | Order MONHYSTERIDA | | |  |  |  |  |  |  |  |  |  |  |  |  |  |  |  |  |  |  |
|  |  | Family AXONOLAIMIDAE | |  |  |  |  |  |  |  |  |  |  |  |  |  |  |  |  |  |  |
|  |  |  | *Axonolaimus* |  |  |  |  |  | √ |  |  |  |  |  |  |  |  |  |  | 1B | 2 |
|  |  |  | *Parodontophora* | √ | √ | √ | √ | √ | √ | √ | √ | √ | √ | √ | √ | √ | √ | √ | √ | 1B | 2 |
|  |  |  | *Pseudolella* |  | √ | √ |  | √ | √ |  |  |  | √ |  |  |  |  |  |  | 1B | 2 |
|  |  | Family DIPLOPELTIDAE | |  |  |  |  |  |  |  |  |  |  |  |  |  |  |  |  |  |  |
|  |  |  | *Campylaimus* |  | √ |  | √ |  | √ |  |  |  |  |  |  |  |  |  |  | 1A | 3 |
|  |  |  | *Southerniella* |  |  |  |  |  | √ | √ |  |  |  |  |  |  |  |  |  | 1B | 3 |
|  |  | Family LINHOMOEIDAE | |  |  |  |  |  |  |  |  |  |  |  |  |  |  |  |  |  |  |
|  |  |  | *Anticyathus* |  |  |  |  | √ | √ |  | √ |  |  |  |  |  |  |  |  | 1B | 3 |
|  |  |  | *Desmolaimus* |  |  |  | √ |  | √ |  |  |  |  |  |  |  | √ |  |  | 1B | 3 |
|  |  |  | *Eumorpholaimus* |  |  | √ |  |  | √ | √ |  | √ | √ |  |  | √ | √ |  |  | 1B | 3 |
|  |  |  | *Linhomoeus* |  |  |  |  |  | √ | √ |  | √ |  |  |  |  |  |  |  | 1B | 3 |
|  |  |  | *Metalinhomoeus* |  |  |  |  |  |  |  |  |  |  | √ |  |  |  |  |  | 1B | 3 |
|  |  |  | *Paralinhomoeus* | √ | √ | √ |  | √ | √ | √ |  |  |  |  |  |  |  |  |  | 1B | 3 |
|  |  |  | *Terschellingia* | √ | √ | √ | √ | √ | √ | √ | √ | √ | √ | √ |  | √ | √ |  |  | 1A | 3 |
|  |  | Family MONHYSTERIDAE | |  |  |  |  |  |  |  |  |  |  |  |  |  |  |  |  |  |  |
|  |  |  | *Diplolaimella* |  |  | √ |  | √ | √ | √ | √ | √ | √ | √ | √ | √ | √ | √ | √ | 1B | 2 |
|  |  |  | *Diplolaimelloides* |  |  |  |  |  |  |  |  | √ |  |  | √ |  | √ | √ | √ | 1B | 2 |
|  |  |  | *Monhystera* |  |  |  |  |  |  |  |  |  |  |  |  |  |  | √ | √ | 1B | 2 |
|  |  |  | *Thalassomonhystera* | √ | √ | √ | √ | √ | √ | √ | √ |  |  |  | √ |  |  | √ |  | 1B | 2 |
|  |  | Family SPHAEROLAIMDAE | |  |  |  |  |  |  |  |  |  |  |  |  |  |  |  |  |  |  |
|  |  |  | *Metasphaerolaimus* |  |  |  |  |  | √ | √ |  |  |  |  |  |  | √ |  |  | 2B | 3 |
|  |  |  | *Parasphaerolaimus* |  | √ | √ | √ |  | √ | √ | √ | √ |  |  |  |  |  |  |  | 2B | 3 |
|  |  |  | *Sphaerolaimus* | √ | √ | √ | √ | √ | √ | √ | √ | √ | √ | √ | √ | √ |  | √ | √ | 2B | 3 |
|  |  | Family XYALIDAE | |  |  |  |  |  |  |  |  |  |  |  |  |  |  |  |  |  |  |
|  |  |  | *Amphimonhystera* | √ | √ |  |  | √ |  |  |  |  | √ |  | √ | √ |  | √ |  | 1B | 2 |
|  |  |  | *Cobbia* |  |  |  |  |  |  |  |  |  |  | √ | √ | √ |  |  | √ | 1B | 2 |
|  |  |  | *Daptonema* | √ | √ | √ | √ | √ | √ | √ | √ | √ | √ | √ |  | √ | √ | √ | √ | 1B | 2 |
|  |  |  | *Elzalia* |  |  |  |  | √ |  |  |  |  |  |  |  |  |  |  |  | 1B | 2 |
|  |  |  | *Linhystera* |  | √ |  | √ |  | √ |  |  |  |  |  |  |  |  |  |  | 1A | 3 |
|  |  |  | *Paramonohystera* |  |  |  |  |  |  |  |  |  |  |  |  |  |  |  | √ | 1B | 2 |
|  |  |  | *Theristus* |  | √ | √ |  | √ | √ |  | √ |  | √ |  |  | √ | √ |  |  | 1B | 2 |
|  | Order ENOPLIDA | | |  |  |  |  |  |  |  |  |  |  |  |  |  |  |  |  |  |  |
|  |  | Family ANOPLOSTOMATIDAE | |  |  |  |  |  |  |  |  |  |  |  |  |  |  |  |  |  |  |
|  |  |  | *Anoplostoma* | √ | √ | √ | √ | √ | √ |  | √ | √ | √ | √ | √ | √ | √ | √ | √ | 1B | 2 |
|  |  | Family ENCHELIDIIDAE | |  |  |  |  |  |  |  |  |  |  |  |  |  |  |  |  |  |  |
|  |  |  | *Calyptronema* |  |  |  |  |  |  |  |  | √ |  | √ | √ | √ | √ |  | √ | 2B | 4 |
|  |  |  | *Polygastrophora* | √ |  |  |  |  | √ |  |  |  |  |  |  |  |  |  |  | 2B | 4 |
|  |  |  | *Symplocostoma* |  |  |  | √ |  |  |  |  |  |  |  |  |  |  |  | √ | 2B | 4 |
|  |  | Family IRONIDAE | |  |  |  |  |  |  |  |  |  |  |  |  |  |  |  |  |  |  |
|  |  |  | *Syringolaimus* |  |  | √ | √ |  | √ |  |  |  | √ | √ |  | √ | √ | √ | √ | 2B | 4 |
|  |  |  | *Trissonchulus* | √ |  | √ |  |  | √ | √ | √ |  |  |  |  |  |  |  |  | 2B | 4 |
|  |  | Family ONCHOLAIMIDAE | |  |  |  |  |  |  |  |  |  |  |  |  |  |  |  |  |  |  |
|  |  |  | *Adoncholaimus* | √ | √ | √ | √ | √ | √ | √ | √ | √ | √ |  | √ |  | √ | √ |  | 2B | 4 |
|  |  |  | *Oncholaimus* |  |  |  |  |  | √ | √ | √ |  |  |  | √ | √ |  | √ |  | 2B | 4 |
|  |  |  | *Viscosia* |  |  |  |  |  |  |  | √ |  | √ |  |  |  |  |  |  | 2B | 3 |
|  |  | Family OXYSTOMINIDAE | |  |  |  |  |  |  |  |  |  |  |  |  |  |  |  |  |  |  |
|  |  |  | *Halalaimus* | √ | √ | √ | √ | √ | √ | √ | √ | √ | √ | √ | √ | √ | √ | √ | √ | 1A | 4 |
|  |  |  | *Oxystomina* | √ |  |  | √ |  | √ | √ |  | √ |  | √ |  | √ | √ |  | √ | 1A | 4 |
|  |  |  | *Thalassoalaimus* | √ | √ |  |  |  | √ | √ |  |  |  |  |  |  |  |  |  | 1A | 4 |
|  |  | Family TRIPYLOIDIDAE | |  |  |  |  |  |  |  |  |  |  |  |  |  |  |  |  |  |  |
|  |  |  | *Bathylaimus* |  |  |  |  |  |  |  |  |  |  |  |  |  | √ |  |  | 2B | 3 |
|  |  |  | *Tripyloides* |  |  | √ |  |  |  |  | √ |  |  |  |  | √ | √ |  | √ | 2B | 3 |
|  | Order DORYLAIMIDA | | |  |  |  |  |  |  |  |  |  |  |  |  |  |  |  |  |  |  |
|  |  | Family ALAIMIDAE | |  |  |  |  |  |  |  |  |  |  |  |  |  |  |  |  |  |  |
|  |  |  | *Amphidelus* |  |  |  |  | √ | √ | √ |  |  |  | √ | √ |  | √ | √ | √ | 1A | 4 |
|  |  | Family APORCELAIMIDAE | |  |  |  |  |  |  |  |  |  |  |  |  |  |  |  |  |  |  |
|  |  |  | *Aporcelaimus* |  |  |  |  |  |  |  |  |  |  |  | √ |  |  |  |  | 2B | 5 |
|  |  | Family DORYLAIMIDAE | |  |  |  |  |  |  |  |  |  |  |  |  |  |  |  |  |  |  |
|  |  |  | *Dorylaimus* |  |  |  |  | √ | √ |  | √ |  |  | √ | √ |  |  | √ | √ | 2B | 4 |
|  |  |  | *Mesodorylaimus* |  |  |  |  |  | √ | √ |  |  |  |  |  |  |  |  |  | 2B | 4 |
|  |  |  | Unidentified Gen. 1. |  |  |  |  |  |  |  | √ |  |  |  |  |  |  |  |  | 2B | 4 |
|  |  | Family LEPTONCHIDAE | |  |  |  |  |  |  |  |  |  |  |  |  |  |  |  |  |  |  |
|  |  |  | *Doryllium* |  |  |  |  |  |  |  | √ |  |  |  | √ |  |  |  |  | 2B | 4 |
|  |  |  | *Proleptonchus* |  | √ |  |  |  | √ |  |  |  |  |  |  |  |  |  |  | 2B | 4 |
|  |  | Family MYLONCHULIDAE | |  |  |  |  |  |  |  |  |  |  |  |  |  |  |  |  |  |  |
|  |  |  | *Mylonchulus* |  |  |  |  |  |  |  | √ |  |  |  |  |  |  |  |  | 2B | 4 |
|  |  | Family NORDIIDAE | |  |  |  |  |  |  |  |  |  |  |  |  |  |  |  |  |  |  |
|  |  |  | *Enchodelus* |  |  |  |  |  |  |  | √ |  |  |  |  |  |  |  |  | 2B | 4 |
|  |  |  | *Lenonchium* | √ |  |  | √ | √ |  |  | √ |  |  |  |  |  |  |  |  | 2B | 4 |
|  |  | Family NYGOLAIMIDAE | |  |  |  |  |  |  |  |  |  |  |  |  |  |  |  |  |  |  |
|  |  |  | *Aquatides* |  |  |  |  |  |  |  | √ |  |  |  |  |  |  |  |  | 2B | 5 |
|  |  |  | *Nygolaimus* |  |  |  |  |  |  |  |  |  |  | √ |  |  |  |  |  | 2B | 5 |
|  |  | Family QUDSIANEMATIDA | |  |  |  |  |  |  |  |  |  |  |  |  |  |  |  |  |  |  |
|  |  |  | *Eudorylaimus* |  |  |  |  |  |  |  |  |  |  |  | √ |  |  |  |  | 2B | 4 |
|  |  |  | *Labronema* |  |  |  |  |  |  |  |  |  |  |  | √ |  |  |  |  | 2B | 4 |
|  |  |  | *Thorneella* |  |  |  |  |  |  |  |  |  |  | √ | √ |  |  | √ | √ | 2B | 4 |
| Class SECERNENTEA | | | |  |  |  |  |  |  |  |  |  |  |  |  |  |  |  |  |  |  |
|  | Order TYLENCHIDA | | |  |  |  |  |  |  |  |  |  |  |  |  |  |  |  |  |  |  |
|  |  | Family CRICONEMATIDAE | |  |  |  |  |  |  |  |  |  |  |  |  |  |  |  |  |  |  |
|  |  |  | *Criconemoides* |  |  |  |  |  |  |  | √ |  |  |  |  |  |  |  |  | Pl | 3 |
|  |  | Family DOLICHODORIDAE | |  |  |  |  |  |  |  |  |  |  |  |  |  |  |  |  |  |  |
|  |  |  | *Dolichodorus* |  | √ |  |  |  | √ | √ | √ |  |  | √ |  |  |  |  |  | Pl | 3 |
|  |  |  | *Tylenchorhynchus* |  |  |  |  |  |  |  | √ |  |  |  |  |  |  |  |  | Pl | 3 |
|  |  | Family HOPLOLAIMIDAE | |  |  |  |  |  |  |  |  |  |  |  |  |  |  |  |  |  |  |
|  |  |  | Unidentified Gen. 1. |  |  |  |  |  |  |  |  |  |  |  | √ |  |  |  |  | Pl | 3 |
|  |  | Family PARATYLENCHIDAE | |  |  |  |  |  |  |  |  |  |  |  |  |  |  |  |  |  |  |
|  |  |  | *Paratylenchus* |  |  |  |  | √ |  |  | √ |  |  | √ | √ | √ |  | √ | √ | Pl | 2 |
|  |  | Family PRATYLENCHIDAE | |  |  |  |  |  |  |  |  |  |  |  |  |  |  |  |  |  |  |
|  |  |  | *Hirschmanniella* |  | √ |  |  |  | √ |  | √ |  |  |  |  |  |  |  |  | Pl | 3 |
|  |  |  | Unidentified Gen. 1. |  |  |  |  |  |  |  |  |  |  |  | √ |  |  | √ | √ | Pl | 3 |
|  |  | Family TYLENCHIDAE | |  |  |  |  |  |  |  |  |  |  |  |  |  |  |  |  |  |  |
|  |  |  | *Tylenchus* |  | √ |  |  | √ | √ |  | √ | √ |  | √ | √ | √ |  |  |  | Pl | 2 |
|  |  |  | Unidentified Gen. 1 |  |  |  |  |  |  |  |  |  |  |  | √ |  |  |  |  | Pl | 2 |
|  |  |  | Unidentified Gen. 2 |  |  |  |  |  |  |  |  |  |  |  | √ |  |  |  |  | Pl | 2 |
|  |  | Family TYLODORIDAE | |  |  |  |  |  |  |  |  |  |  |  |  |  |  |  |  |  |  |
|  |  |  | *Cephalenchus* |  |  | √ |  |  |  |  |  |  |  | √ | √ |  | √ |  | √ | Pl | 2 |
|  | Order APHELENCHIDA | | |  |  |  |  |  |  |  |  |  |  |  |  |  |  |  |  |  |  |
|  |  | Family SEINURIDAE | |  |  |  |  |  |  |  |  |  |  |  |  |  |  |  |  |  |  |
|  |  |  | *Seinura* |  |  |  |  |  |  |  |  |  |  |  | √ |  |  | √ | √ | 2B | 2 |
|  | Order RHABDITIDA | | |  |  |  |  |  |  |  |  |  |  |  |  |  |  |  |  |  |  |
|  |  | Family CEPHALOBIDAE | |  |  |  |  |  |  |  |  |  |  |  |  |  |  |  |  |  |  |
|  |  |  | *Eucephalobus* |  |  |  |  |  |  |  |  |  |  |  | √ |  |  |  |  | 1B | 2 |
|  |  | Family DIPLOGASTRIDAE | |  |  |  |  |  |  |  |  |  |  |  |  |  |  |  |  |  |  |
|  |  |  | *Mononchoides* |  |  |  |  |  |  |  |  |  |  | √ | √ |  | √ | √ |  | 1B | 1 |
|  |  | Family PANAGROLAIMIDAE | |  |  |  |  |  |  |  |  |  |  |  |  |  |  |  |  |  |  |
|  |  |  | *Panagrolaimus* |  |  |  |  |  |  |  |  |  |  |  | √ |  |  | √ |  | 1B | 1 |
|  |  | Family RHABDITIDAE | |  |  |  |  |  |  |  |  |  |  |  |  |  |  |  |  |  |  |
|  |  |  | *Mesorhabditis* |  |  |  |  |  |  |  |  |  |  |  | √ |  |  | √ |  | 1B | 1 |
|  |  |  | *Rhabditis* |  |  |  |  |  | √ |  | √ |  |  | √ |  |  |  |  | √ | 1B | 1 |
|  |  |  | Unidentified Gen. 1 |  |  |  |  |  |  |  |  |  |  |  | √ |  |  |  |  | 1B | 1 |
|  |  |  | Unidentified Gen. 2 |  |  |  |  |  |  |  |  |  |  |  | √ |  |  |  |  | 1B | 1 |

Appendix S2. Results of model selection using Akaike’s information criterion (AIC) for the relationship between nematode taxonomic diversity, phylogenetic diversity, proportion of species in each feeding group, colonizer-persister values and environmental factors (see Table 2 for abbreviations). The information-theoretic Akaike’s information criterion corrected for small samples (AICc), change in AICc relative to the top-ranked model (AICc), AICc weight (wAICc = model probability) and R^2^ values as a measure of the model’s goodness-of-fit. Only the top three models are shown.

| **Models** | | **AIC*_c_*** | **ΔAIC*_c_*** | ***w*AIC*_c_*** | **R^2^** | **Models** | **AIC*_c_*** | | **ΔAIC*_c_*** | | ***w*AIC*_c_*** | **R^2^** |  |  |
| --- | --- | --- | --- | --- | --- | --- | --- | --- | --- | --- | --- | --- | --- | --- |
| Genus richness | |  |  |  |  | Species richness | | |  | |  |  |  |  |
| AT AP ATR | | 302.19 | 0 | 0.24 | 0.32 | AT AP ATR | 313.86 | | 0 | | 0.25 | 0.42 |  |  |
| AT AP ATR NDVI | | 304.45 | 2.26 | 0.08 | 0.33 | AT AP ATR NDVI | 315.58 | | 1.72 | | 0.10 | 0.43 |  |  |
| AT AP ATR TN% | | 304.51 | 2.32 | 0.08 | 0.33 | AT AP ATR TC% | 316.04 | | 2.18 | | 0.08 | 0.43 |  |  |
| *H’* at species level | |  |  |  |  | *H’* at genus level | | |  | |  |  |  |  |
| AT AP ATR | | 66.06 | 0 | 0.12 | 0.31 | AT ATR | | 63.34 | 0 | | 0.13 | 0.25 |  |  |
| AT ATR | | 66.82 | 0.76 | 0.08 | 0.26 | AT AP ATR | | 63.83 | 0.48 | | 0.11 | 0.28 |  |  |
| AT AP ATR Sand% | | 67.20 | 1.13 | 0.07 | 0.33 | AT ATR TN% | | 65.33 | 1.99 | | 0.05 | 0.26 |  |  |
| AvTD | |  |  |  |  | AvPD | | |  | |  |  |  |  |
| pH | | 295.10 | 0 | 0.05 | 0.09 | AT AP ATR | 282.79 | | 0 | | 0.09 | 0.40 |  |  |
| ATR | | 295.22 | 0.12 | 0.05 | 0.09 | AT AP ATR NDVI | 282.91 | | 0.12 | | 0.08 | 0.43 |  |  |
| AT | | 295.39 | 0.30 | 0.05 | 0.08 | AT AP NDVI | 283.92 | | 1.13 | | 0.05 | 0.39 |  |  |
| Proportion of species of *c-p* 3-5 | | |  |  |  | Proportion of individual of *c-p* 3-5 | | | | |  |  |  |  |
| AT TC% | 375.64 | | 0 | 0.05 | 0.31 | AT ATR pH | | 435.77 | | 0 | 0.08 | 0.42 |  |  |
| AT TC% pH | 375.76 | | 0.13 | 0.05 | 0.34 | AT ATR TC% TN% pH | | 435.85 | | 0.08 | 0.08 | 0.48 |  |  |
| ATR TC% pH | 376.58 | | 0.94 | 0.03 | 0.33 | AT ATR TC% TN% pH NDVI | | 436.07 | | 0.30 | 0.07 | 0.50 |  |  |
| Proportion of species of *c-p* 2 | | |  |  |  | Proportion of individual of *c-p* 2 | | | | |  |  |  |  |
| AT TC% | | 368.80 | 0 | 0.05 | 0.26 | AT ATR | 434.11 | | | 0 | 0.11 | 0.35 |  |  |
| TC% pH NDVI | | 368.90 | 0.10 | 0.05 | 0.30 | AT ATR pH | 434.61 | | | 0.50 | 0.08 | 0.37 |  |  |
| AT TC% pH | | 369.61 | 0.81 | 0.03 | 0.29 | AT ATR TN% | 435.49 | | | 1.38 | 0.05 | 0.36 |  |  |
| Proportion of species of selective deposition feeders | | | | |  | Proportion of individual of selective deposition feeders | | | | | |  |  |  |
| AT | | 357.67 | 0 | 0.05 | 0.18 | AP TN% pH | 409.30 | | | 0 | 0.09 | 0.48 |  |  |
| AT ATR | | 358.09 | 0.42 | 0.04 | 0.21 | AT ATR TN% pH | 409.72 | | | 0.42 | 0.07 | 0.51 |  |  |
| AT NDVI | | 358.13 | 0.45 | 0.04 | 0.21 | AP pH | 410.39 | | | 1.09 | 0.05 | 0.45 |  |  |
| Proportion of species of non-selective deposition feeders | | | | |  |  | | | | |  |  |  |  |
| pH | | 353.86 | 0 | 0.08 | 0.17 |  |  | | |  |  |  |  |  |
| AP pH | | 355.50 | 1.64 | 0.03 | 0.18 |  |  | | |  |  |  |  |  |
| AT NDVI | | 355.59 | 1.74 | 0.03 | 0.18 |  |  | | |  |  |  |  |  |

Appendix S3. Non-metric multidimensional scaling (MDS) ordinations of nematode communities from (a) 16 locations and (b) three vegetation types. The location of each sampling location is described in Table 1.

Appendix S4. Results from BIO-ENV analysis of soil nematode communities and environmental variables. The best combination of environment variables includes all variables. Correlation coefficients are given for the best combination of environmental variables and for the best single environmental variable (ATR: Annual temperature range; NDVI: Yearly normalised difference vegetation index).

| No. of variables | Correlation | Variable Selections |
| --- | --- | --- |
| 1 | 0.590 | ATR |
| **2** | **0.615** | **ATR, pH** |
| 3 | 0.614 | ATR, pH, NDVI |
